# Supplementary material for: The Protective Effects of Iron Free Lactoferrin on Lipopolysaccharide-Induced Intestinal Inflammatory Injury via Modulating the NF-κB/PPAR Signaling Pathway
Source: Foods. 2022 Oct 26;11(21):3378. doi: 10.3390/foods11213378 (PMC9658706; doi:10.3390/foods11213378)
Supplement: Supplementary file 1 [file foods-11-03378-s001.zip › foods-1946901-supplementary.pdf]

Supplementary figures

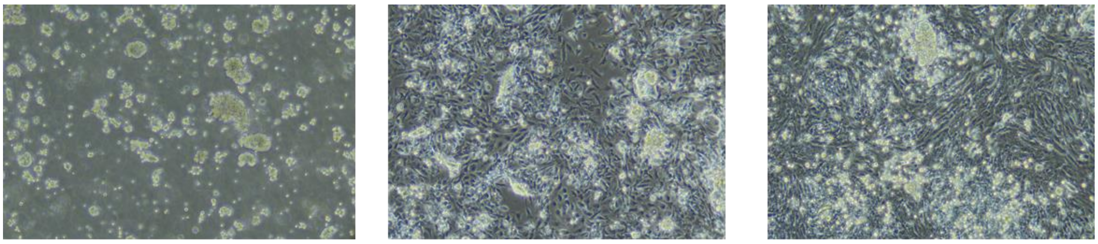

1h 24h 48h

Figure S1. The cell morphology was observed at 1 h, 24 h and 48 h under microscope.

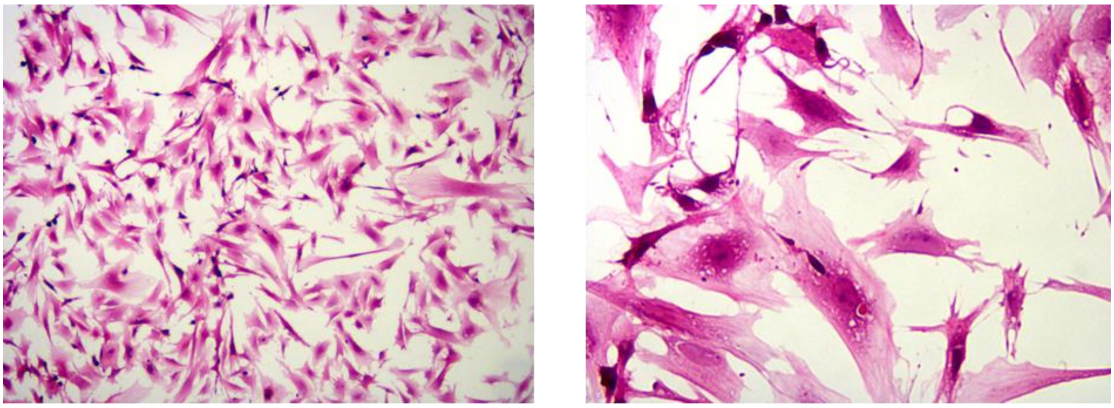

100× 400×

Figure S2. After HE staining, the cell morphology was observed 100 times and 400 times under the microscope.

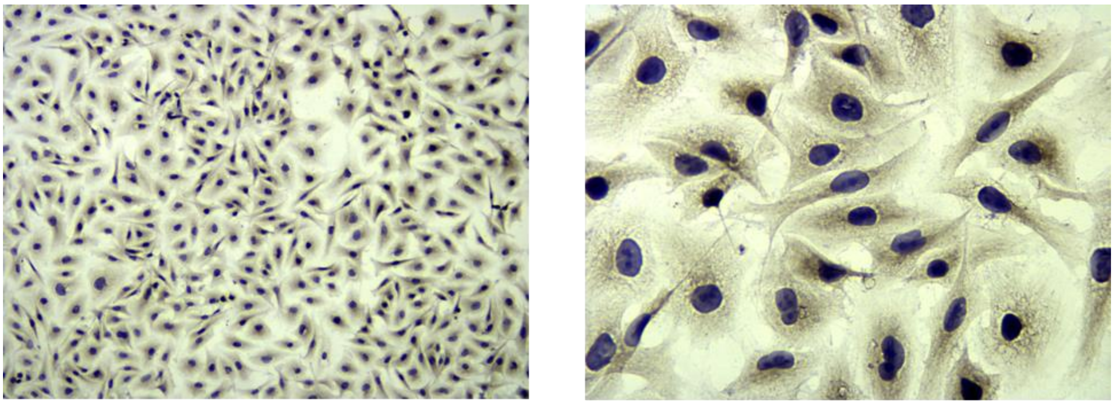

100× 400×

Figure S3. After immunohistochemistry, the cell morphology was observed using the microscope.

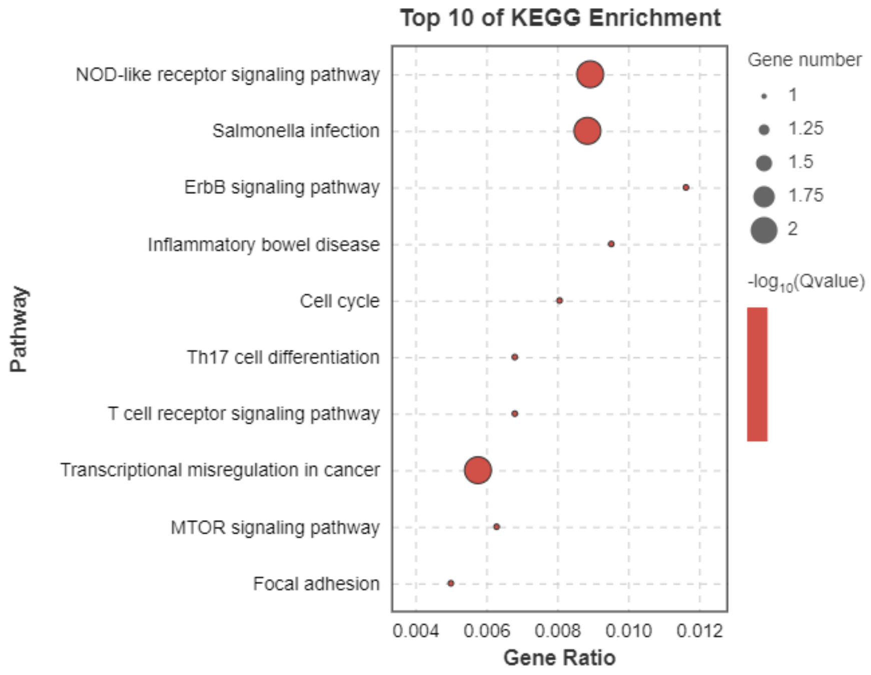

Figure S4. The top 10 inflammation-related KEGG pathways enriched by the103 unique DEGs in the combined group of LF + LPS compared with LPS.

Supplementary tables

Table S1 The list of primer sequences.

Genes	Forward (5'- 3')	Reverse (5'- 3')
Tnfaip3	CAATATGAGGAAAGCGGTGAAG	GGATGATCTCTCGAAACTGTGG
Nfkbia	TGGAAGTGATTGGTCAGGTGA	AGGCAAGATGGAGAGGGGTAT
Relb	CCCCTACAATGCTGGCTCCCTGAA	CACGGCCCGCTCTCCTTGTTGATT
Scd2	CTTTGTCGCTGAGGTCTGAA	TTACTCAGCCACCACCACA
Bcl3	CCGGAGGCCCTTTACTACCA	GGAGTAGGGGTGAGATGGCAG
Ccl2	CTTCTGGGCCTGCTGTTCA	CCAGCCTACTCATTGGGATCA
GAPDH	TCAAGAAGGTGGTGAAGCAG	AAGGTGGAAGAGTGGGAGTTG

Table S2 The list of 52 DEGs related to inflammation in the four treatment groups.

Symbol	control-1_fpkm	control-2_fpkm	control-3_fpkm	LPS-1_fpkm	LPS-2_fpkm	LPS-3_fpkm	LF-1_fpkm	LF-2_fpkm	LF-3_fpkm	LF-LPS-1_fpkm	LF-LPS-2_fpkm	LF-LPS-3_fpkm
Lck	0.29	0.58	0.38	1.24	0.8	0.91	0.83	0.74	0.86	0.67	0.83	0.82
Pparg	1.1	1.2	0.84	0.24	0.44	0.42	0.55	0.54	0.58	0.42	0.67	0.51
Ppard	8.76	10.65	9.17	21.04	17.14	19.41	19.77	16.79	17.52	18.39	14.87	16.15
Angptl4	60.8	47.54	52.4	23	21.4	19.5	26.92	24.75	28.26	18	15.5	17.87
Relb	7.27	5.81	6.62	18.53	17.58	17.29	17.2	16.4	18.73	16.82	16.18	15.78
Lbp	1.98	1.6	1.68	9.43	9.7	9.11	12.57	11.78	12.45	11.21	9.81	9.16
Cd40	3.5	2	2.88	1.27	1.53	1.35	1.47	1.53	1.65	0.96	1.23	1.5
Ccl4	1.05	0.97	0.86	2.77	2.94	3.71	3.46	2.32	2.22	3.4	1.61	2.35
Tnfaip3	3.89	5.05	3.55	12.02	13.7	12.49	12.83	10.39	12.8	12.43	11.99	11.6
Acsl6	0.47	0.64	0.78	0.36	0.26	0.22	0.3	0.28	0.28	0.29	0.23	0.41
Nfkbia	14.13	13.43	15	52.98	51.39	51.97	48.24	49.74	46.73	52.14	46.24	51.12
Fos	4.56	5.42	4.77	41.11	41.2	38.27	56.48	57.52	50.64	47.99	51.4	51.07
Map3k8	1.5	1.34	1.37	5.92	6.85	7.02	7.56	7.46	7.03	6.87	8.61	8.4
Tnf	0.24	0.2	0.28	0.98	0.9	1.07	0.75	0.65	0.48	1.34	0.99	1.52
Scd2	103.26	105.89	104.54	289.54	294.48	306.21	231.57	210.74	224.43	283.85	273.45	274.06
Il6	4.77	7.08	7.1	15.73	18.93	16.13	18.73	26.43	19.44	15.24	21.76	16.9
Il1r1	15.52	17.2	17.08	42.8	48.86	45.82	55.08	56.14	47.1	45.97	49.21	46.44
Tnfrsf11a	1.07	1.06	0.94	0.38	0.35	0.4	0.42	0.3	0.26	0.26	0.39	0.39
Pfkfb3	16.68	19.32	18.92	15.17	15.68	15.72	21.37	17.48	17.29	14.25	13.37	13.65
Jag1	14.86	16.51	15.54	6.58	6.46	6.74	7.18	7.12	5.8	6.68	6.46	6.29
Il1b	0	0	0	0.87	0.71	0.9	0.5	0.39	0.54	0.29	0.15	0.07

Hmgcs2	0.41	0.32	0.59	0	0.14	0.09	0.08	0.05	0.14	0.06	0.09	0.21
Vcam1	28.94	31.18	31.19	81.98	96.91	94.97	89.22	93.99	79	79.94	87.38	83.91
Tnfrsf1b	17.8	16.92	16.52	39.78	35.87	37.28	37.36	34.57	37.78	36.84	32.05	36.81
Cxcl5	1.64	2.68	1.04	25.14	32.78	29.11	34.04	39.58	33.72	22.94	25.72	22.18
Cxcl3	0.24	0.41	0.5	7.56	7.96	10.31	6.5	6.5	4.49	4.61	4.73	5.07
Cxcl1	1.35	1.59	1.19	38.56	41.26	39.99	50.62	53.77	49.35	32.78	39.42	33.51
Olr1	5.26	6.07	4.47	11.72	13.19	12.13	12.99	14.38	13.43	14.86	18.44	12.63
Vegfd	20.53	20.4	19.96	60.49	61.52	63.38	67.47	69.82	66.72	62.65	59.04	63.66
Il15	0.22	0.63	1.03	0.21	0.18	0.29	0.31	0.15	0.08	0.21	0.18	0.36
Birc3	3.64	4.89	4.83	7.99	10.36	9.91	8.43	8.16	7.94	8.54	10.57	8.5
Acsbg1	0.48	0.73	0.59	0.06	0.02	0.1	0.13	0.04	0.04	0.07	0.05	0.03
Plcg2	2.37	1.86	1.8	1.09	0.85	0.83	0.67	0.93	0.94	1.03	0.65	0.95
Lif	2.46	2.57	2.4	8.93	8.3	8.41	11.09	10.24	10.28	6.93	6.45	6.85
Cxcl10	1.08	1.3	1.96	4.65	4.64	4.79	4.34	4.96	4.4	6.15	4.54	4.39
Ccl2	21.39	23.2	21.54	172.28	199.56	208.25	138.7	170.75	148.05	135.58	157.32	152.33
Scd1	14.19	17.7	15.59	44.42	48.85	49.17	32.97	30.11	30.14	42.56	42.46	43.22
Icam1	10.85	9.88	10.12	22.57	19.56	20.86	20.05	19.7	21.03	20.91	18.32	20.94
Mmp3	0.44	0.38	0.31	1.58	1.2	1.42	1.06	1.08	1.08	1.05	0.9	0.97
Mapk10	0.29	0.34	0.3	0.15	0.13	0.13	0.12	0.19	0.21	0.12	0.29	0.13
Cd14	2.11	1.73	2.41	9.43	8.37	8.62	6.09	6.32	6.7	6.65	6.17	6.64
Socs3	11.53	11.45	10.27	35.58	33.34	35.65	31.76	31.57	33.5	33.51	29.96	33.45
Bcl3	7.87	8.27	7.45	34.59	33.42	35.03	30.57	32.43	31.06	30.7	28.77	31.53
Cebpb	25.66	23.8	36.58	91.55	80.33	94.68	81.38	96.5	87.59	67.75	58.48	67.82
Cxcl2	0.29	0.17	0.1	3.7	4.8	5.78	3.86	3.01	2.84	3.74	4	3.05
Fabp4	2.93	5.02	5.11	11.21	12.47	13.06	10.74	12.37	10.64	10.29	13.34	13.16

Tgtp2	5.34	4.25	5.28	1.97	3.1	2.11	2.42	2.85	2.44	2.58	2.41	2.7
Cpt1b	0.45	0.41	0.48	0.28	0.04	0.09	0.13	0.12	0.17	0.35	0	0.38
Hmgcs1	22.53	29.11	26.82	63.49	72.36	68.57	53.16	55.19	48.51	64.89	83.38	73.17
Ccl21a	3.64	3.2	2.31	0.25	0.59	0.72	0.55	0.32	1.14	0.22	0.27	0.63
Ccl21b	0.29	0.06	0.37	0	0	0	0	0	0.3	0	0	0
Gm10591	0.29	0.06	0.37	0	0	0	0	0	0.3	0	0	0
